# The 9th annual computational and systems neuroscience (cosyne) meeting

**DOI:** 10.1186/2042-1001-2-3

**Published:** 2012-03-30

**Authors:** Agnieszka Grabska-Barwińska, Cindy Poo

**Affiliations:** 1 Gatsby Computational Neuroscience Unit, UCL, London, UK; 2 Champalimaud Neuroscience Programme, Lisbon, Portugal

## Abstract

The 9th annual Computational and Systems Neuroscience meeting (Cosyne) was held 23–26 February in Salt Lake City, Utah. Cosyne meeting is the forum for exchange of experimental and theoretical/computational approaches to studying systems neuroscience.

## Main text

The 9th annual Computational and Systems Neuroscience meeting (Cosyne) was held 23 to 26 February in Salt Lake City, Utah (abstracts are freely available online: http://cosyne.org/cosyne12/Cosyne2012_program_book.pdf). Cosyne responds to a growing demand for exchange between experimental and theoretical approaches to systems neuroscience. To facilitate interdisciplinary interactions, it is organized in a single track, comprising 43 invited and contributed talks. These are enriched by open-ended poster sessions starting late in the evening, allowing for more informal and detailed discussions (297 posters over three poster sessions). There is an additional two days of workshops, which takes place after the main meeting at the Snowbird ski resort. The success of Cosyne over the years is demonstrated by the steady growth in abstract submissions (with the record number of over 520 this year).

The opening talk of the meeting corroborated the transparency of Cosyne organization and the abstract selection process. Jonathan Pillow, co-chair of the program committee, explained in detail mechanisms employed to maximize fairness of the selection procedure. It was an ice-breaking presentation, demonstrating that theory need not be boring. He reported on the review process and demonstrated how sophisticated data models can entertainingly expose surprising statistical effects. Having used Generalized Linear Model to analyze submitted abstracts, he provided a list of words with the most “influence” on having the work accepted (among most “helpful” were: “responses”, “optimal”, “activity”) or rejected (cf. “simulations”, “data”, “spike”).

Posters can serve as a good proxy of trends and hot topics in neuroscience. Of all sensory systems, vision still remains the most widely studied (40 posters, vs. 10 per each of other sensory systems). In cognition, decision-making and reward were the best represented topics (32, compare to an average of 5 per attention, memory, objects and categories). There was an increase in the representation of motor and behavioral studies (16 posters) in comparison to previous years, reflecting growing interest in these areas. There was also a noticeably greater focus on dynamical and/or latent variable models for explaining multi-electrode data, as these kinds of data sets have become more available. Overall, Cosyne this year has seen a positive growth in the representation of experimental and systems neuroscience. We only highlight a few talks, trying to reflect the spectrum of topics important to Cosyne in 2012.

## Cognitive systems

Rebecca Saxe (Massachusetts Institute of Technology) presented her work on representing mental experience of others. She discussed development of the Theory of Mind, exemplified by entertaining movies of a 3- year old failing to and a 5-year old correctly inferring other people’s experiences and thoughts. Several cortical regions involved in the Theory of Mind were delineated, as supported by fMRI imaging and rTMS interference studies. Multi-voxel pattern analysis provided additional information on how spatial pattern of activity changes when thinking of physical vs. emotional states. Recently, Saxe’s group performed similar analysis in congenitally blind people, indicating that they construct a valid theory of sight, as opposed to developing simple compensative mechanisms.

Tom Griffiths (University of California, Berkeley) described recent attempts in merging statistics, machine learning and human cognition. Probabilistic distribution of human behavior seems to be consistent with the distribution resulting from Bayes rule. In reality, exact Bayesian inference is rarely possible and must be replaced by approximate algorithms, such as Monte Carlo methods. Several machine learning algorithms (i.e., importance sampling, particle filtering) have been explored as potential mechanisms for human inference. Recognizing the constraints of human cognition, a novel algorithm (a sequential Monte Carlo scheme based on the “win-stay, lose-shift” principle) was proposed. 

Bayesian theory of cognition was next challenged by Bruno Averbeck (National Institute of Mental Health) whose behavioral data argue that evidence seeking in humans is less than optimal. In their experiment, participants viewed sequences of color beads drawn from a hidden urn. After they were shown each bead, they could either choose to draw another bead from the urn or finish the task by inferring the majority bead color in the urn. Comparison with a Bayesian model revealed that participants drew fewer samples than optimal. Also, in difficult tasks they increased evidence sampling, but not as much as the optimal Bayesian model would predict. This suboptimality was most evident in participants characterized as impulsive, as well as in Parkinson patients with dopamine-induced impulse control disorders.

## Sensory systems: Vision

Jeremy Freeman (New York University) gave an exciting account on how theoretical models of natural images (from Eero Simoncelli’s lab) guided experimental studies and the understanding of visual cortex function. From a hierarchical model of image structure, he derived a set of stimuli differing in complexity (i.e. correlations of visual features, such as orientations, frequencies and positions). Both electrophysiological findings in monkeys and fMRI data from humans indicated increased specialization of V2 — a visual area downstream from the striate cortex. While V1 response did not reflect the change in the correlational structure of an image, while V1 responses to images with or without higher-order correlation structures were similar, V2 activity in response to the presence or absence of these higher-order structures were distinguishable.

Najib Majaj from Jim DiCarlo’s group (MIT) gave a provocative talk, where he claimed that the neural population codes in monkey IT outperform any computer vision system in explaining human visual object recognition (discriminability of images that explore shape similarity and identity preserving image). Interestingly, the match to the human psychophysical data could only be obtained from IT neuronal populations, and not V4 neurons.

## Sensory systems: Audition

Sarah Woolley (Columbia University) spoke about neural coding schemes in three stages of auditory processing in the songbird zebra finch. Their group observed a change from the dense coding scheme at the midbrain to a sparse and highly selective coding scheme at primary and higher forebrain auditory regions. Individual neurons become highly selective to the target signal embedded in a specific complex auditory scene, showing “non-classical” spectrotemporal receptive fields of auditory neurons. Woolley also presented a model which accounts for the encoding of song from dense to sparse, capturing the non-linear spatiotemporal receptive field transformation across several stages of auditory processing.

## Sensory systems: Olfaction

Recent rise of interest in the olfactory system was apparent at Cosyne through the number of invited speakers on this topic, a two-day dedicated workshop at Snowbird and a number of posters.

Zachary Mainen presented work from his group (Champalimaud Neurosciences Programme) examining the origins and uses of uncertainty in odor-guided decisions. Two main sources of variability in rats’ reaction times were identified: the sensory system noise (leading to “speed-accuracy tradeoff” in an odor discrimination task) and much slower fluctuations explained by online (trial-to-trial) reinforcement learning (discovered in an odor categorization task). Fluctuations due to online reinforcement learning seemed to influence the behavioral strategy chosen by rats: in the discrimination task rats clearly increased odor sampling time with task difficulty whereas no benefits (or use) of such an increase was observed in the categorization task. Thus, the post-decision wagering of behavioral outcome seems to limit the benefits of increased odor sampling time.

## Motor systems

Sources of behavioral variability were brought up again by Kris Chaisanguanthum from Philip Sabes’ group (University of California, San Francisco). Chaisanguanthum examined the variability in arm movements in relation to noise in motor planning activity of dorsal premotor cortex (PMd) and primary motor cortex (M1). They reported two types of variability in reach movement data. The first was a “fast” source of variability around the mean for speed and initial direction. An additional “slow” drift exists on the time course of minutes, accounting for a large portion of overall variance (20-40%). Interestingly, the slow variability can be well-predicted by neural population activity in PMd and M1, whereas the “fast” noise seems to be largely independent of neural activity. The authors suggest that the slower, longer time scale drift may be the result of noise in a continuous online learning process that is centrally generated and inherent to the generation of voluntary motor movements. This is reminiscent of the slow trial-to-trial variability in odor categorization by rats (see above, Mainen’s talk), also attributed to reinforcement learning. Thus, across systems, tasks and animal models, we can see convergence of interpretations of phenomena, up until quite recently disregarded as “just noise”.

In an elegant study, Ben Dongsung Huh from Terry Sejnowski’s group (Salk Institute and University of California, San Diego) derived a novel conservation principle that predicts a constant level of drive for human arm movements. They extended the optimal control models to predict movement durations and angular speed for reaching and circle drawing movements in humans. Their predictions were later confirmed in experiments where subjects made unconstrained hand movements. The simple and elegant principle suggested by this study provides a fresh perspective on neural computation in motor pre-planning of basic human movements.

## Memory

Shantanu Jadhav from Loren Frank’s group (University of California, San Francisco) examined the relationship between sharp-wave ripples in the hippocampus and spatial working memory. Hippocampal sharp-wave ripples sharp-wave ripples are suggested to be important for spatial memory consolidation and retrieval. However, no direct evidence has demonstrated this. Jadhav showed that transient suppression of hippocampal activity during sharp-wave ripples in rats causes a marked learning deficit specific to spatial working memory with no discernible effect on spatial reference memory. These novel results demonstrate that place cells are not sufficient to support working spatial memory, and awake replay of past experience during sharp wave ripples is an essential component of working memory processes that allow an animal to retrieve specific memories and use them to guide behavior.

## Novel methods: Optogenetics

Following the increase in numbers of symposia and posters “optogenetics” at the annual Society for Neuroscience (SfN) meetings for the past several years, this year at Cosyne optogenetics was given a full session.

Mehrdad Jazayeri presented novel results from a collaboration with Gregory Horwitz (University of Washington), demonstrating channelrhodopsin-2 (ChR2) elicitted behavioral responses in rhesus monkeys. The use of optogenetic tools in non-human primates has been limited. Jazayeri and colleagues show that activation of ChR2 in neurons of primary visual cortex (V1) produced phosphenes at the location of the corresponding receptive fields as tested in their behavioral paradigm. Monkeys were trained to either maintain fixation on a central target, or saccade to a visual target after the fixation point offset. Optical stimulation was delivered in half of the trials of each type. On trials with optical stimulation but no visual target presentation, monkeys made saccades towards the receptive fields of neurons at the ChR2 injection site. Simultaneous recording from neurons nearby the infection site show that optical stimulation had variable effects on firing rates.

As in previous years, Cosyne 2012 proved to be an energetic and focused forum for the exchange of innovative ideas and methods for computational and systems neuroscience. The steadily rising number of submissions and participants demonstrates a strong need for a meeting that seeks to highlight both novel experimental approaches and the development of new theoretical ideas. Cosyne provides an evermore-valuable venue for experimental and computational neuroscientists to meet and grow together.

Looking ahead, a relocation of the Cosyne meeting venue seems possible, as participants responded enthusiastically to an informal poll for holding Cosyne in Europe. 2013 will be the 10-year anniversary of Cosyne. It will be a great opportunity to survey progress in the field since the inception of the meeting, while discussing challenges that still lie ahead.

## Abbreviations

ChR2, Channelrhodopsin-2; fMRI, Functional magneti resonance imaging; rTMS, Repetitive transcranial magnetic stimulation; PMs, Dorsal premotor cortex; M1, Primary motor cortex; V1, Primary visual cortex; V2, Secondary visual cortex; IT, Inferior temporal cortex.

## Competing interest

The authors declare they have no competing financial interests. CP is a postdoctoral fellow in Zachary Mainen’s lab.

## Authors’ contributions

Authors are listed in an alphabetical order; their contributions to writing the manuscript were equal. Both authors read and approved the final manuscript.

